# Why don't people with MCI approach memory clinics? The role of awareness in medical help-seeking

**DOI:** 10.3389/fneur.2022.897737

**Published:** 2022-08-24

**Authors:** Ariela Gigi, Merav Papirovitz

**Affiliations:** Department of Psychology, Ariel University, Ariel, Israel

**Keywords:** Alzheimer's disease, Mild Cognitive Impairment, anxiety, subjective memory complaints (SMC), meta-cognition, early diagnosis, memory decline, help-seeking

## Introduction

Early as possible diagnosis of Alzheimer's Disease (AD) has some critical implications: it provides the possibility for the patient and his close relatives to prepare for what's to come (e.g., educate the future caregiver), minimize potential risks [e.g., driving; (1)] and enables them to seek for symptomatic treatment (2). For these reasons, the importance of diagnosing patients at the “Mild Cognitive Impairment” (MCI, a pre-clinical stage of AD) phase is clear. The current paper aims to briefly review how impaired awareness might impede early diagnosis and to offer a way to handle this challenge.

## Early diagnosis and help-seeking behavior

Early diagnosis is dependent on active help-seeking. The answer to why some people seek help in memory clinics and others do not is complicated: it was already argued that objective cognitive impairment does not necessarily constitute the main explanation for consultation (3). This is likely the reason no significant differences were found in objective memory abilities between help-seekers (HS) in memory clinics and non-help seekers (NHS) (4, 5). However, HS exhibited more subjective memory complaints (SMC) and described their memory deterioration as worse than NHS, regardless of their objective memory ability (4, 6).

Studies have shown that SMC are often more associated with anxiety than with objective memory deficits [e.g., (7)]. In a 10-year long-term study, the researchers compared participants who perceived themselves as very anxious with people who perceived themselves as having low anxiety. The high-stress group rated their memory abilities as deteriorating over time and reported increasing failures in their memory abilities. These results were obtained even though no objective differences in memory abilities were found between the two groups (8). Similar results were also obtained when physiological manifestations of anxiety were examined: a study in healthy adults found that participants with SMC had higher urinary cortisol levels than participants without SMC, regardless of objective cognitive performance (9). Linking these findings to help-seeking behavior, Gigi et al. (4) found that HS exhibited more SMC and higher anxiety levels from memory deterioration compared to NHS. This outcome was in the absence of differences in objective memory measures between these two groups (HS vs. NHS).

Yet, an acceptable notion is that most elders with SMC (regardless of their objective memory status) do not seek medical help (10). The question that should be asked is why do most MCI individuals do not approach memory clinic?

## Awareness of cognitive and emotional state

The decision to seek help requires awareness of the cognitive changes, and moreover, an evaluation that help is needed (1). In the same manner, the ability to detect this cognitive deterioration and the accompanying anxiety sensations requires self-awareness. However, recent studies found that participants with MCI may exhibit reduced awareness of the memory deterioration and their emotional state: regarding cognitive awareness, it was found that MCI participants exhibit poor awareness of their episodic memory abilities (11). Another study demonstrated reduced emotional awareness among MCI individuals (12). The latest compared objective and subjective measures of anxiety by assessing changes in skin conductivity (as an indication of objective anxiety) while administering memory task. In addition, an anxiety questionnaire (as an indication of subjective anxiety) was used immediately after completing the memory task. It was found that although MCI participants exhibited elevation in physiological arousal during the memory task, they didn't report enhanced anxiety. The authors proposed that this reduction in awareness may result in not approaching professional help and, hence, missing early diagnosis.

## Discussion

If the objective cognitive condition is not the primary reason for help-seeking (3), then who are the people that do approach memory clinics? Regardless of their memory condition, what distinguishes HS from NHS is (1) HS have more SMC; (2) they are more likely to perceive their cognitive deficits as causing concern; (3) they perceive their cognitive deficits as a biomedical cause (13); (4) HS report of higher levels of anxiety which is directly related to their cognitive condition (4) (5) report of more symptoms of depression (6). Clearly, perceiving the above requires awareness as a necessary condition. Thus, we suggest that MCI individuals who seek for professional help are characterized with intact awareness of their cognitive and emotional state.

It should be taken into consideration that the issue of awareness among MCI participants is complicated: studies assessing cognitive awareness among MCI are not conclusive, varying from reporting on intact memory awareness to anosognosia of their cognitive deficits (14–17). Comprehension of the emotional awareness among MCI is also limited, but only a few studies refer to this matter (12, 18, 19). We suggest that among individuals with MCI, the factor distinguishes between help seekers and non-help seekers is the ability to be aware of their cognitive deterioration and to their anxiety from this decline. A recent review paper found a decline in aspects of awareness in the course of deterioration from MCI to dementia (20). Thus, we suggest that although various factors might affect help-seeking behavior, an intact awareness is a key component for approaching memory clinics. This awareness is expected to deteriorate as the disease progresses.

The importance of our conception also relies on the findings that impaired awareness may be used as a predictor for AD pathology among people with MCI: it was found that people who underestimated their cognitive decline exhibit poorer performance in memory tasks, smaller hippocampus volume, and worse AD pathology (compared to people that overestimated their decline) (21). If limited awareness of cognitive and emotional state characterizes people with MCI, it is likely that they will not go to memory clinics and, as a result, will not be diagnosed in the early stages of the disease. Since early detection of this group is critical for early diagnosis, we recommend raising awareness among primary care physicians regarding the complexity of the situation and deepening the attention to changes in patients' memory, even if they are not worried or anxious.

## Author contributions

AG obtained the main ideas of this opinion article and revised the manuscript for accurate intellectual content. MP drafted the manuscript and contributed to the expression of the idea. All authors contributed to the article and approved the submitted version.

## Conflict of interest

The authors declare that the research was conducted in the absence of any commercial or financial relationships that could be construed as a potential conflict of interest.

## Publisher's note

All claims expressed in this article are solely those of the authors and do not necessarily represent those of their affiliated organizations, or those of the publisher, the editors and the reviewers. Any product that may be evaluated in this article, or claim that may be made by its manufacturer, is not guaranteed or endorsed by the publisher.
